# Temporal Regulation of Natural Killer T Cell Interferon Gamma Responses by β-Catenin-Dependent and -Independent Wnt Signaling

**DOI:** 10.3389/fimmu.2018.00483

**Published:** 2018-03-16

**Authors:** Jessica C. Kling, Margaret A. Jordan, Lauren A. Pitt, Jana Meiners, Thao Thanh-Tran, Le Son Tran, Tam T. K. Nguyen, Deepak Mittal, Rehan Villani, Raymond J. Steptoe, Kiarash Khosrotehrani, Stuart P. Berzins, Alan G. Baxter, Dale I. Godfrey, Antje Blumenthal

**Affiliations:** ^1^The University of Queensland Diamantina Institute, Translational Research Institute, Brisbane, QLD, Australia; ^2^Comparative Genomics Centre, James Cook University, Townsville, QLD, Australia; ^3^Department of Microbiology and Immunology, Peter Doherty Institute for Infection and Immunity, University of Melbourne, Parkville, VIC, Australia; ^4^Fiona Elsey Cancer Research Institute; and Federation University, Ballarat, VIC, Australia; ^5^ARC Centre of Excellence in Advanced Molecular Imaging, University of Melbourne, Parkville, VIC, Australia

**Keywords:** Wnt, β-catenin, natural killer T cell, α-galactosylceramide, interferon gamma, IL-4, IL-12

## Abstract

Natural killer T (NKT) cells are prominent innate-like lymphocytes in the liver with critical roles in immune responses during infection, cancer, and autoimmunity. Interferon gamma (IFN-γ) and IL-4 are key cytokines rapidly produced by NKT cells upon recognition of glycolipid antigens presented by antigen-presenting cells (APCs). It has previously been reported that the transcriptional coactivator β-catenin regulates NKT cell differentiation and functionally biases NKT cell responses toward IL-4, at the expense of IFN-γ production. β-Catenin is not only a central effector of Wnt signaling but also contributes to other signaling networks. It is currently unknown whether Wnt ligands regulate NKT cell functions. We thus investigated how Wnt ligands and β-catenin activity shape liver NKT cell functions *in vivo* in response to the glycolipid antigen, α-galactosylceramide (α-GalCer) using a mouse model. Pharmacologic targeting of β-catenin activity with ICG001, as well as myeloid-specific genetic ablation of *Wntless (Wls)*, to specifically target Wnt protein release by APCs, enhanced early IFN-γ responses. By contrast, within several hours of α-GalCer challenge, myeloid-specific *Wls* deficiency, as well as pharmacologic targeting of Wnt release using the small molecule inhibitor IWP-2 impaired α-GalCer-induced IFN-γ responses, independent of β-catenin activity. These data suggest that myeloid cell-derived Wnt ligands drive early Wnt/β-catenin signaling that curbs IFN-γ responses, but that, subsequently, Wnt ligands sustain IFN-γ expression independent of β-catenin activity. Our analyses in ICG001-treated mice confirmed a role for β-catenin activity in driving early IL-4 responses by liver NKT cells. However, neither pharmacologic nor genetic perturbation of Wnt production affected the IL-4 response, suggesting that IL-4 production by NKT cells in response to α-GalCer is not driven by released Wnt ligands. Collectively, these data reveal complex temporal roles of Wnt ligands and β-catenin signaling in the regulation of liver NKT cell activation, and highlight Wnt-dependent and -independent contributions of β-catenin to NKT cell functions.

## Introduction

Natural killer T (NKT) cells are an important population of hepatic lymphocytes in both humans and mice (1). These innate-like lymphocytes orchestrate immune responses against viral and bacterial infections, as well as during carcinogenesis, autoimmunity, injury, and fibrosis (2, 3). NKT cells respond rapidly to glycolipid antigens presented by the MHC-I-like molecule CD1d, in conjunction with co-stimulation *via* CD40 on antigen-presenting cells (APCs) with CD40L on NKT cells (4). Antigen presentation by APCs and recognition by NKT cells, as well as CD40/CD40L ligation elicit cytokine production by both APCs (e.g., IL-12) and NKT cells [interferon gamma (IFN-γ), IL-4, IL-17], among other cellular responses (4, 5). The concerted actions of these cytokines determine the flavor of NKT cell contributions to immune responses in the liver environment.

Hepatic Wnt proteins are central regulators of cell proliferation, differentiation, and functionality during liver injury, repair, regeneration, as well as homeostasis (6, 7). Their roles are complex and often context dependent. More recently, Wnt ligands have emerged as important regulators of immune responses during infection, cancer, and autoimmunity (8–10). The 19 mammalian Wnt proteins engage receptors of the Frizzled (Fzd) family, together with co-receptors including low-density lipoprotein receptor-related proteins (LRP) 5/6, receptor tyrosine kinase-like orphan receptor (Ror), and receptor-like tyrosine kinase (Ryk) (11). Palmitoylation of Wnt proteins by the acyltransferase Porcupine in the endoplasmic reticulum, as well as subsequent binding to the chaperone Wntless (Wls), are required for the functionality and release of most Wnt proteins from secreting cells (12–14). Depending on the nature of the Wnt/Wnt receptor complex, Wnt proteins activate cells *via* β-catenin-dependent or -independent signaling pathways. In the absence of Wnt ligation, casein kinase-1 and glycogen synthase kinase-3β phosphorylate β-catenin within the β-catenin destruction complex, which also contains the scaffold proteins adenomatous polyposis coli and axis inhibition (Axin). Phosphorylated β-catenin is targeted for proteasomal degradation (15). Wnt/receptor engagement inactivates the destruction complex, stabilizes β-catenin, and enables its nuclear translocation, where it acts as a coactivator for transcription factors of the T cell factor (TCF)/lymphoid enhancing factor (LEF) family (15). By contrast, β-catenin-independent signaling comprises different pathways, including the Wnt/Ca^2+^, JNK, and planar cell polarity pathways, which govern cytoskeletal rearrangements and cell polarization (11). Wnt signaling is highly regulated by soluble factors including Wnt inhibitory factor (Wif), Dickkopf (Dkk) family members, and soluble Frizzled-related proteins (sFRPs) (16).

β-Catenin has been implicated in directing NKT cell development and functions (17). LEF-1-binding sites are present in the human *CD1D* promoter, and LEF-1 negatively regulates *CD1D* expression (18, 19). In mice, conditional knockout of β-catenin decreases thymic NKT cell numbers, in contrast to increases in NKT cell numbers upon transgenic β-catenin overexpression. In these experiments, IL-4- and IL-17-expressing NKT cell subsets were primarily affected (20). Moreover, differentiation of IL-4-producing NKT cells in the periphery was governed by Lef-1 activity (21). Stimulation of β-catenin-overexpressing NKT cells *in vitro* and *in vivo* resulted in decreased IFN-γ expression, and increased IL-4, IL-13, and IL-17 production, consistent with the effects on the development of NKT cell subsets in these mice (20). These observations strongly suggest key roles for β-catenin and its interaction partners, TCF and LEF, in the development and functions of NKT cells.

The complex phenotypical changes in genetically targeted mouse models have thus far made it difficult to separate effects on NKT cell development from functional effects on NKT cells upon antigen encounter. Furthermore, whether Wnt ligands are directly involved in the governance of NKT cell functions *in vivo* remains unknown. Evidence that Wnt ligands regulate T cell responses, at least in part by modulating functions of APCs (22, 23), led us to hypothesize that Wnt proteins exert similar regulatory functions on NKT cell activation. We thus employed pharmacologic and genetic perturbation of Wnt production and β-catenin to investigate how β-catenin activity and Wnt ligands shape NKT cell cytokine responses *in vivo* using the model antigen, α-galactosylceramide (α-GalCer).

## Materials and Methods

### Mice

C57BL/6 mice were bred in house or obtained from the Animal Resources Centre (Perth, WA, Australia). Tcf/Lef TOPFlash reporter mice, which express luciferase as a reporter of Tcf/Lef transcriptional activity (24), were bred to obtain mice heterozygous for the transgene or wild-type littermate controls. Conditional *Wls* knockout mice (*Wls*^fl/fl^ LysM-Cre^+^) and their littermate controls (*Wls*^fl/fl^ LysM-Cre^−^) were obtained by backcrossing *Wls*^fl/fl^ mice (25) with C57BL/6 mice for 10 generations, and subsequent cross-breeding with mice expressing Cre recombinase driven by the Lysozyme M promoter (LysM-Cre) (7), backcrossed for at least 7 generations with C57BL/6. Mice were housed under specific pathogen-free conditions with food and water *ad libitum*. All procedures involving animals adhered to the guidelines of the National Health and Medical Research Council Australian Code for the Care and Use of Animals for Scientific Purposes, and were approved by the Animal Ethics Committee of The University of Queensland (UQDI/571/12; UQDI/554/15) and the University of Melbourne Animal Ethics Committee (06089).

Male and female mice aged 8–14 weeks were housed in microisolator cages (Technoplast) with corncob bedding, nesting material, and houses. Mice were injected intravenously, during the light cycle, with 2 µg α-GalCer (KRN7000; Funakoshi Company), or an equivalent volume of vehicle control (5.6% sucrose/0.75% l-histidine/0.5% Tween 20 in PBS). ICG001 (20 mg/kg; Tocris Bioscience) and IWP2 (20 mg/kg; Tocris Bioscience), or an equivalent volume of DMSO (Sigma-Aldrich) as a vehicle control, were diluted in PBS and injected intraperitoneally 16 h prior to α-GalCer injection.

### Flow Cytometry

To obtain single cell suspensions, liver tissues were mechanically disrupted, cells pelleted, and resuspended in 7.5 mL PBS and 4.5 mL isotonic Percoll (Sigma-Aldrich), and centrifuged at room temperature for 15 min at 760 × *g*. Leukocytes and red blood cells (RBCs) were harvested, and RBCs lysed in lysis buffer (0.15 M NH_4_Cl, 1 mM KHCO_3_, 0.1 mM EDTA). Intracellular cytokine staining was performed on leukocytes using the Cytofix/Cytoperm kit (BD Biosciences) according to the manufacturer’s instructions. Fc receptor binding was blocked with anti-mouse CD16/32 (2.4G2; BD Pharmingen) before addition of antibodies against mouse CD3 (145-2C11 or 17A2; PE, PerCP or eFluor 450; eBioscience), NK1.1 (PK136; APC; eBioscience and BD Pharmingen), Fzd1 (162531; biotin; R&D Systems), Fzd7 (151143; biotin; R&D Systems), TCRβ (H57-597; BV421; BioLegend), CD11b (M1/70; PE; BD Pharmingen), Ly-6G (1A8; Alexa Fluor 700; BioLegend), Ly-6C (AL-21; PE-Cy7; BD Biosciences), IFN-γ (XMG1.2; FITC; BD Pharmingen), and IL-4 (BVD4-1D11; PE; BD Pharmingen). Streptavidin-PE-Cy7 (BD Pharmingen) was used for detection of biotinylated antibodies. α-GalCer-loaded CD1d tetramer (PE) was generated in-house using a baculovirus expression system, similar to that previously described (26). Samples were acquired on a BD FACSCanto or Beckman Coulter Gallios and analyzed using FlowJo software (TreeStar, Inc.).

### Bone Marrow-Derived Macrophages

Bone marrow cells from femurs and tibias of male and female mice aged 8–14 weeks were cultured in DMEM (Life Technologies) containing 10% FBS (Bovogen Biologicals), 2 mM l-glutamine, 1 mM sodium pyruvate, 10 mM HEPES (all Life Technologies), and 20% L cell-conditioned medium. Fresh medium (one-fifth of original volume) was added every second day. After 6 days, adherent cells were harvested by washing with warm PBS, followed by incubation with cold 0.1 M EDTA in PBS for 10 min at 4°C. Cells were washed with cold PBS, centrifuged, and resuspended in TRIzol (Life Technologies).

### Quantitative Real-time PCR

RNA was isolated from liver tissue using TRIzol, and 1 µg total RNA was reverse transcribed with the iScript cDNA synthesis kit (Bio-Rad Laboratories). Quantitative real-time PCR was performed with SYBR Green PCR master mix (Life Technologies) using an ABI Prism 7900HT (PerkinElmer). Expression of genes of interest was normalized to the housekeeping gene hypoxanthine guanine phosphoribosyl transferase (*Hprt*) [2^(Ct value^ *^Hprt^*^ − Ct value gene of interest)^] and is depicted as relative gene expression. Gene-specific primers were designed using the Integrated DNA Technologies online primer design tool or were previously published (Table 1).

**Table 1:** List of primers used in quantitative PCR.

Gene	Forward primer (5′-3′)	Reverse primer (5′-3′)	Reference
*Hprt*	GCCCCAAAATGGTTAAGGTTGC	AACAAAGTCTGGCCTGTATCCAAC	
*Cd1d*	CCTATTTGTCCGTGGTCTCC	ACAGGTTTTGGGTAGAAGCC	
*Cd40*	CTGTGAGGATAAGAACTTGGAGG	AGAGAAACACCCCGAAAATGG	
*Dkk1*	ATATCCCAGAAGAACCACACTG	ATCTTGGACCAGAAGTGTCTTG	
*Ifng*	GAACTGGCAAAAGGATGGTGA	TGTGGGTTGTTGACCTCAAAC	
*Il12b*	GGAAGCACGGCAGCAGAATA	AACTTGAGGGAGAAGTAGGAATGG	(27)
*Il4*	CGAATGTACCAGGAGCCATATC	TCTCTGTGGTGTTCTTCGTTG	
*Luciferase*	GGCGCGTTATTTATCGGAGTT	GTTGAGCAATTCACGTTCAT	
*Sfrp1*	AATGTGACAAGTTCCCCGAG	GATGGCCTCTGACTTCAACTC	
*Tbx21*	CAACAACCCCTTTGCCAAAG	TCCCCCAAGCAGTTGACAGT	(28)
*Wnt1*	GATTTTGGTCGCCTCTTTGG	CGTGGCATTTGCACTCTTG	
*Wnt2*	GTAGATGCCAAGGAGAGGAAAG	CCAGCATGTCCTCAGAGTAC	
*Wnt2b*	GCCCTCATGAACTTACACAAC	CTGTGCGTCGGAAGTCTG	
*Wnt3*	CCCGCTCAGCTATGAACAAG	ACTTTAGGTGCATGTGGTCC	
*Wnt3a*	GTGAGGACATTGAATTTGGAGG	ACTTGAGGTGCATGTGACTG	
*Wnt4*	AGTGCCAATACCAGTTCCG	AGAGATGGCGTATACAAAGGC	
*Wnt5a*	CGCTAGAGAAAGGGAACGAATC	CTCCATGACACTTACAGGCTAC	
*Wnt5b*	GACTGACGCCAACTCCTG	TGCTCCTGATACAACTGACAC	
*Wnt6*	TCAAGACTCTTTATGGATGCGC	ATGGCACTTACACTCGGTG	(7)
*Wnt7a*	ACGAGTGTCAGTTTCAGTTCC	AATCGCATAGGTGAAGGCAG	
*Wnt7b*	AATGAGGCGGGCAGAAAG	TGCGTTGTACTTCTCCTTGAG	
*Wnt8a*	TCATGTACGCAGTCACCAAG	TTTTCCCCGAACTCCACG	
*Wnt8b*	GTACACCCTGACTAGAAACTGC	AAACTGCTTGGAAATTGCCTC	
*Wnt9a*	GGGACAACCTCAAGTACAGC	TTCCACTCCAGCCTTTATCAC	
*Wnt9b*	CCAAGAGAGGAAGCAAGGAC	AACAGGTACGAACAGCACAG	
*Wnt10a*	CGCTTCTCTAAGGACTTTCTGG	GTGGCATTTGCACTTACGC	
*Wnt10b*	TCTCGGGATTTCTTGGATTCC	CATTTGCACTTCCGCTTCAG	
*Wnt11*	CCAAGCCAATAAACTGATGCG	GGCATTTACACTTCGTTTCCAG	
*Wnt16*	CGAGAGGTGGAACTGTATGG	TGAATGCTGTCTCCTTGGTG	

### ELISA

Mouse IFN-γ (R&D Systems) and IL-12p40 (BD Biosciences) ELISA kits were used according to the manufacturer’s protocols to determine cytokine concentrations in mouse serum. Serum concentrations of IL-4 were determined using a sandwich ELISA. Maxisorp plates (Nunc) were coated with anti-mouse IL-4 capture antibody (2 µg/mL; 11B11; BioLegend) in 50 mM sodium bicarbonate buffer (pH 9.4) overnight. Plates were washed with 0.05% Tween 20 (Sigma-Aldrich) in PBS, blocked with 4% bovine serum albumin (BSA; Roche) in PBS for 2 h, and then samples and standards (mouse rIL-4; Peprotech), serially diluted two-fold from 2,000 pg/mL, were added for 1 h. Anti-mouse IL-4 detection antibody (0.5 µg/mL; BVD6-24G2, BioLegend) diluted in 4% BSA in PBS was added for 1 h, followed by HRP-conjugated streptavidin (2 µg/mL; Sigma-Aldrich) for 30 min. The plates were developed with TMB substrate (BD Biosciences), and the reaction stopped with 1 M H_2_SO_4_ (Sigma-Aldrich).

### Microarray Analysis

Natural killer T cells were purified from liver lymphocytes by flow cytometric cell sorting as α-GalCer-CD1d-tetramer^+^ TCRβ^+^ NK1.1^+^ cells and further divided on the basis of CD4 expression. The purity of CD4^−^ and CD4^+^ NKT cell subsets analyzed by flow cytometry post-sort was >96%. Sorted NKT cell subsets (four samples/group) were stimulated with Dynabeads Mouse CD3/CD28 T Cell Expander Beads (Invitrogen) at a 1:1 ratio of beads to cells for 2 h at 37°C. RNA was extracted from stimulated NKT cell subsets (Qiagen RNeasy and Qiashredder), RNA yield quantified spectrophotometrically on a Nanodrop ND-1000 and aliquots electrophoresed for determination of sample concentration and purity. Microarray hybridizations were performed using 100 ng RNA and the WT Expression kit (Life Technologies), WT Terminal Labeling and Controls Kit (Affymetrix), and Affymetrix Mouse Gene_1.0ST arrays (770,317 probe sets representing an estimated 35,556 mouse transcripts). The probed arrays were washed and stained using the GeneChip Hybridization Wash and Stain Kit (Affymetrix), and scanned using the GeneChip Scanner 3000. Images (.dat files) were processed using GeneChip Command Console (Affymetrix) and CEL files imported into Partek Genomics Suite 6.6 (Partek SG) for further analysis as published previously (29, 30).

### Statistical Analyses

One- or two-way ANOVA with Dunnett’s or Bonferroni’s correction for multiple comparisons, or unpaired two-sided Student’s *t*-test were performed as indicated using Graphpad Prism (Graphpad Software, Inc.). *P* values <0.05 were considered statistically significant.

## Results

### α-GalCer Rapidly Induces Dynamic Regulation of IFN-γ and IL-4 Responses in the Liver and Serum

**Figure 1:**
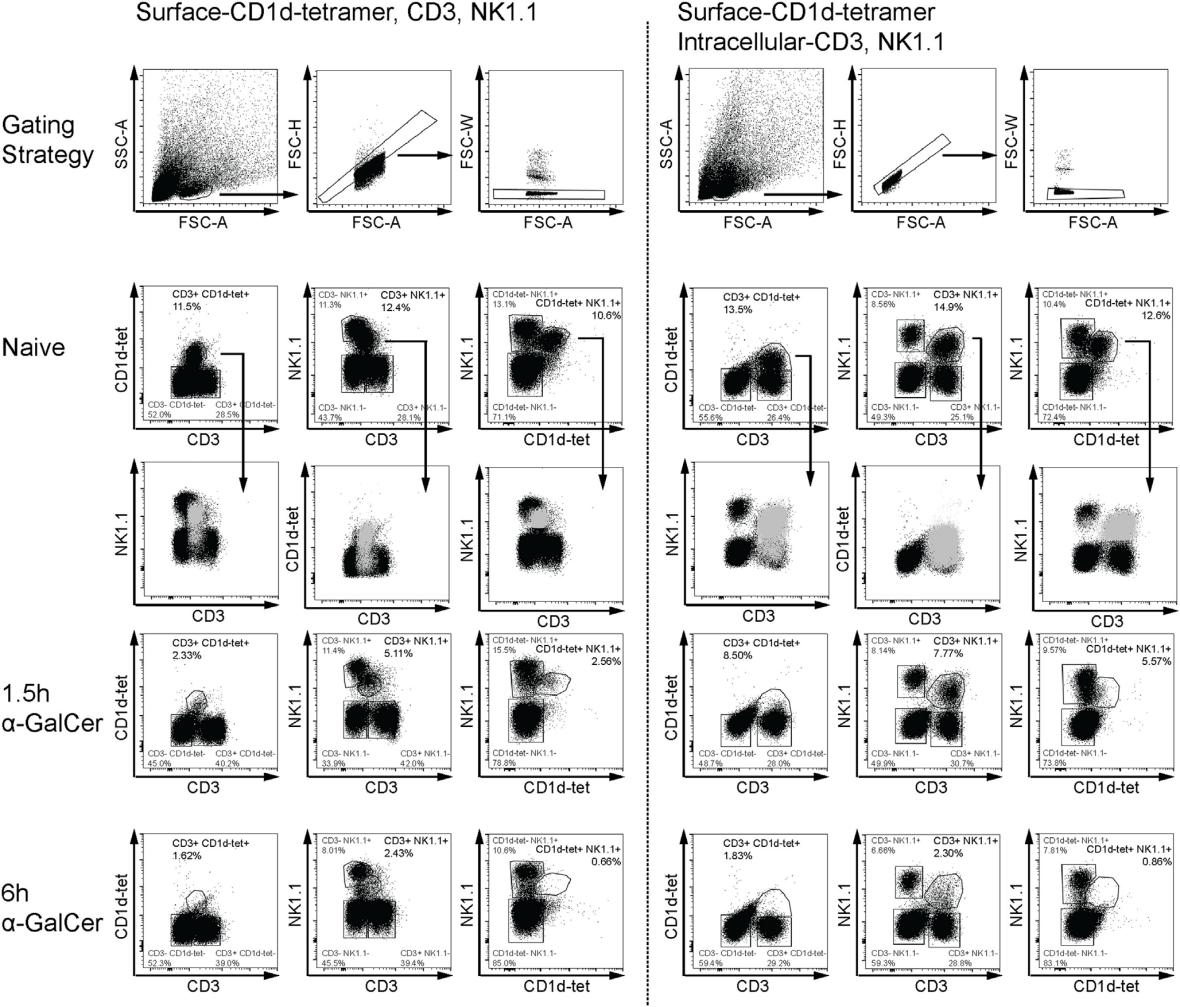
Flow cytometry-based detection of liver natural killer T (NKT) cells. Liver leukocytes from naïve or α-GalCer-challenged mice were stained with surface CD1d tetramer (CD1d-tet). Half of the liver sample was stained for surface expression of CD3 and NK1.1 (left panel), while the other half was permeabilized for intracellular expression of CD3 and NK1.1 (right panel). Various gating strategies for CD3, NK1.1, and CD1d-tet expression over time are shown as a comparison of surface and intracellular expression. Putative “NKT” cells from naïve mice (light gray) have been overlayed to show placement within dot plots using the third marker. Surface expression of all three markers are reduced as early as 1.5 h after α-galactosylceramide (α-GalCer) challenge (left panel); however, intracellular CD3 and NK1.1 expression is sustained at this early time point (right panel). Data are representative of three mice per time point.

α-Galactosylceramide rapidly induces IFN-γ and IL-4 expression by NKT cells (5), accompanied by transient downregulation of several distinguishing surface markers (31). To reliably detect liver CD3^+^NK1.1^+^ NKT cells in α-GalCer-challenged mice, we established an intracellular flow cytometry protocol for CD3 and NK1.1 to track NKT cells *in vivo* early after α-GalCer challenge (Figure 1). Consistent with previous reports (31), CD3, NK1.1, and CD1d tetramer surface staining was reduced after α-GalCer challenge (left panel). As CD1d tetramer was unable to enter cells upon permeabilization, antibodies against CD3 and NK1.1 were used for intracellular staining. Importantly, while NKT cells exhibit dim CD3 surface staining, they have similar staining intensity of intracellular CD3 as CD3^+^NK1.1^−^ T cells (right panel). This allows for better separation of the CD3^+^NK1.1^+^ population from other cell populations, particularly after challenge. At 1.5 h after challenge, a greater proportion of CD3^+^NK1.1^+^ was detected using the intracellular staining protocol, compared to surface only staining. However, similar percentages of CD3^+^NK1.1^+^ cells were detected at 6 h using either staining protocol, indicating that these cells have either degraded CD3 and NK1.1, or are no longer present in liver tissue.

**Figure 2:**
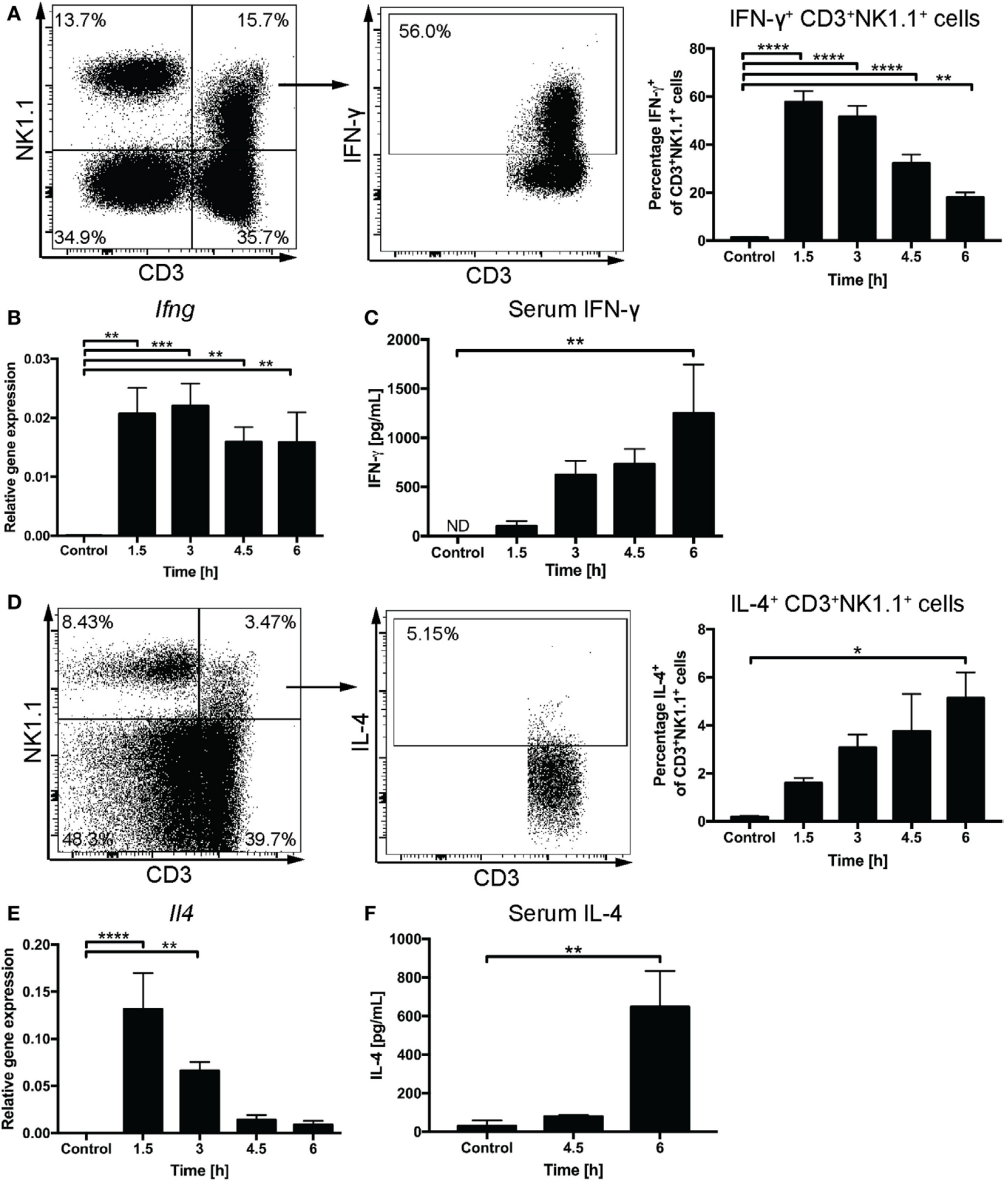
Rapid induction of interferon gamma (IFN-γ) and IL-4 expression upon α-galactosylceramide (α-GalCer) challenge. Percentages of CD3^+^NK1.1^+^ natural killer T cells in the liver expressing IFN-γ **(A)** or IL-4 **(D)** were determined by flow cytometry. Representative plots for IFN-γ are from 1.5 h post challenge, while IL-4 expression is demonstrated for 6 h post challenge. Quantitative PCR was performed to determine mRNA expression of *Ifng* **(B)** and *Il4* **(E)** in liver tissue upon α-GalCer challenge. Serum concentrations of IFN-γ **(C)** or IL-4 **(F)** were determined by ELISA. ND, not detected. *n* = 4–7 mice per time point from two independent experiments. One-way ANOVA with Dunnett’s correction for multiple comparisons; *p < 0.05, **p < 0.01, ***p < 0.001, ****p < 0.0001. [Notes **(D,F)**: mice received a DMSO-injection prior to α-GalCer challenge; **(F)**: 6-h time point replicated in Figure 4B.]

We used the intracellular staining method to follow IFN-γ and IL-4 expression by liver CD3^+^NK1.1^+^ cells after systemic administration of α-GalCer. A rapid increase of IFN-γ^+^CD3^+^NK1.1^+^ cells with 57.8 ± 4.6% cells expressing IFN-γ was observed at 1.5 h and 51.6 ± 4.6% at 3 h post challenge (Figure 2A). By contrast, the proportion of IFN-γ^+^CD3^+^NK1.1^−^ cells peaked at 9.7 ± 1.6% at 1.5 h after challenge (Figure S1A in Supplementary Material) suggesting that at these early time points, the majority of cells expressing IFN-γ in response to α-GalCer challenge are CD3^+^NK1.1^+^ NKT cells. This was accompanied by a significant increase in liver *Ifng* mRNA expression (Figure 2B) and gradually increasing concentrations of IFN-γ in the serum (Figure 2C). While the percentage of CD3^+^NK1.1^+^ cells expressing IFN-γ declined to 32.2 ± 3.7 and 18.0 ± 2.1% at 4.5 and 6 h post challenge, respectively, the *Ifng* mRNA expression as well as IFN-γ serum concentrations remained elevated (Figures 2A–C). This is likely reflective of secondary contributions by IFN-γ-producing CD3^−^NK1.1^+^ NK cells (Figure S1B in Supplementary Material).

The percentage of liver CD3^+^NK1.1^+^ cells expressing IL-4 gradually increased over the first 6 h after α-GalCer challenge allowing for IL-4 detection in the serum by 6 h. This was preceded by a transient upregulation of liver *Il4* mRNA expression that peaked at 1.5 h after α-GalCer challenge (Figures 2D–F). A similar percentage of CD3^+^NK1.1^−^ cells and CD3^+^ NK1.1^+^ cells expressed IL-4, which may be attributable to the presence of NK1.1^−^ NKT cells (32) (Figure S1C in Supplementary Material).

### Suppression of β-Catenin Activity Facilitates IFN-γ Expression but Limits IL-4 Responses after α-GalCer Challenge *In Vivo*

**Figure 3:**
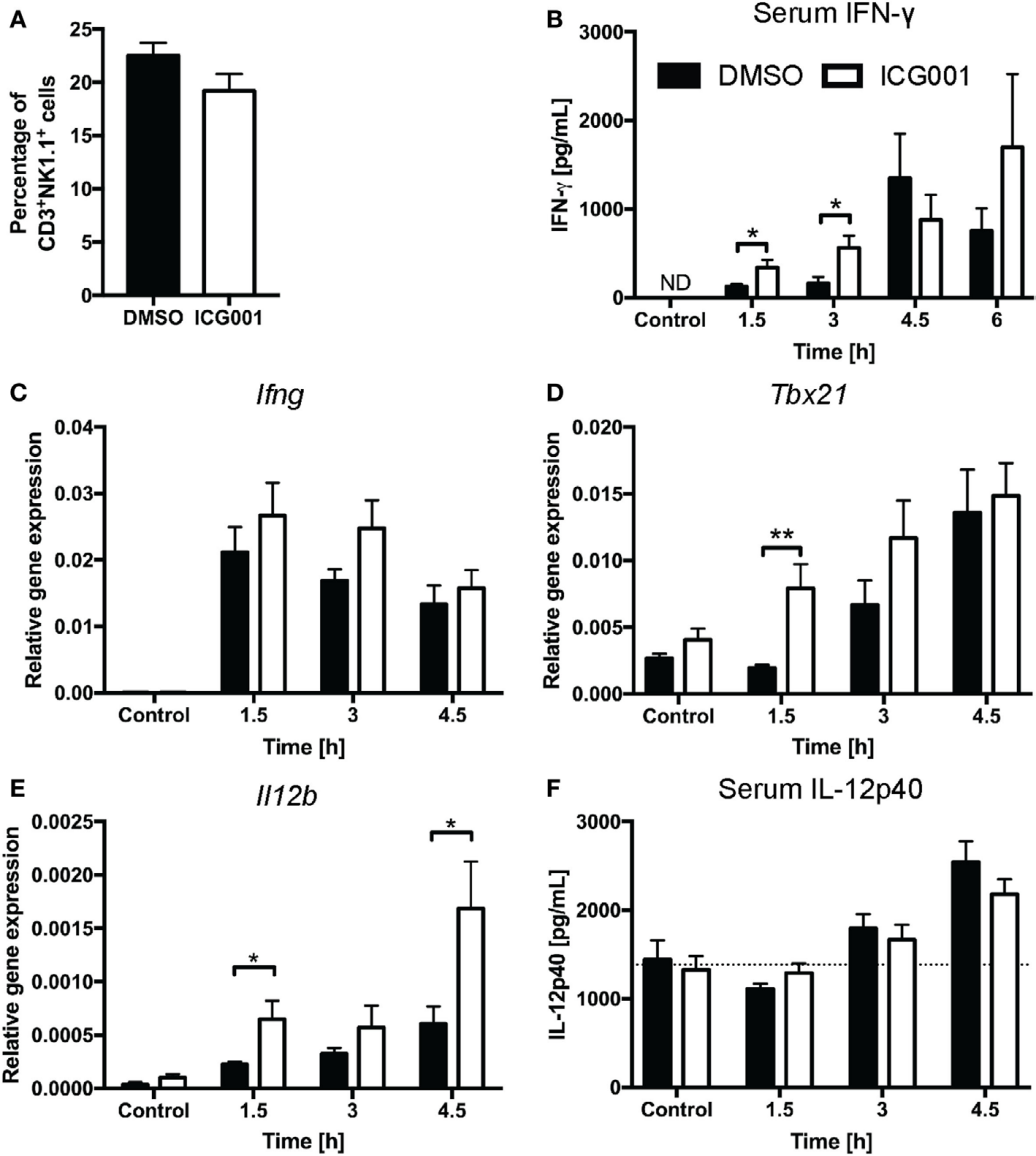
β-catenin activity suppresses the IL-12/interferon gamma (IFN-γ) axis upon α-galactosylceramide (α-GalCer) challenge. **(A)** Percentages of CD3^+^NK1.1^+^ natural killer T cells in liver tissue after overnight challenge with ICG001, an inhibitor of β-catenin activity, or DMSO. After α-GalCer challenge, **(B)** serum concentrations of IFN-γ were determined by ELISA. The mRNA expression of *Ifng* **(C)** and *Tbx21* **(D)** in the liver was determined by quantitative RT-PCR. **(E)** mRNA expression of *Il12b* in the liver and **(F)** protein expression of IL-12p40 in the serum. Dotted line represents average concentration of serum IL-12p40 for all mock-injected mice. Data are means ± SEM from four to nine mice per treatment for each time point cumulative from two to three independent experiments. Groups were compared by unpaired *t* test at each time point; *p < 0.05, **p < 0.01, N.D., not detectable.

**Figure 4:**
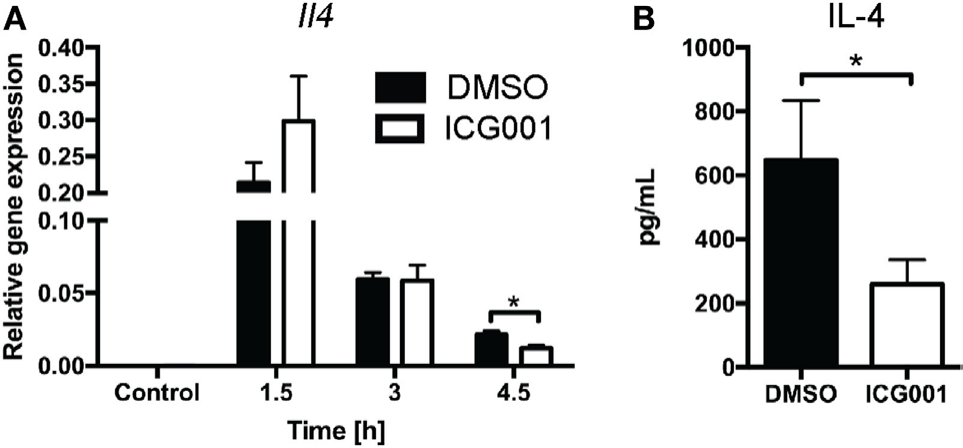
β-catenin activity promotes IL-4 expression upon α-galactosylceramide (α-GalCer) challenge. Mice were treated with ICG001, then challenged with α-GalCer. **(A)** The mRNA expression of *Il4* in the liver was determined by quantitative PCR. **(B)** Serum concentrations of IL-4 at 6 h after α-GalCer challenge. Data are means ± SEM from four to nine mice per treatment for each time point analyzed cumulatively in two to three independent experiments. Groups were compared by *t*-test at each time point; *p < 0.05.

Previous studies have indicated that β-catenin contributes to NKT cell development, particularly affecting IL-4-expressing NKT cells (20, 21). As genetic modulation of β-catenin expression affects NKT cell development *in vivo* (20, 21), we chose a small molecule inhibitor approach to assess the role of β-catenin activity in antigen-driven NKT cell activation in mice with normal NKT cell development. ICG001 inhibits β-catenin interaction with CREB-binding protein (CBP) (33) and has been successfully used to modulate β-catenin functions in mouse models of fibrosis and cholangiocarcinoma (34, 35). Importantly, and in contrast to the changes in NKT cell proportions seen in studies with genetically altered β-catenin expression throughout NKT cell development, ICG001 did not alter the percentages of CD3^+^NK1.1^+^ NKT cells in naïve mice or after α-GalCer challenge (Figure 3A and data not shown). ICG001-treated mice had increased serum IFN-γ concentrations at 1.5 and 3 h after α-GalCer challenge (Figure 3B), which was accompanied by a trend toward elevated liver *Ifng* mRNA expression and more rapid induction of *Tbx21* mRNA (encoding for the transcription factor driving *Ifng* expression) (Figures 3C,D). At these early timepoints, the major contributor to IFN-γ expression is the CD3^+^NK1.1^+^ NKT cell population (Figures S1A,B in Supplementary Material). Interferon gamma expression by NKT cells drives IL-12 responses. mRNA expression of *Il12b* was significantly higher in liver tissue of ICG001-treated mice compared to DMSO controls (Figure 3E). This effect of ICG001 may be restricted to the liver environment, as serum levels of IL-12p40 were comparable between ICG001- and DMSO-treated mice (Figure 3F). Similar to *Il12b*, liver mRNA expression of *Cd40*, a costimulatory molecule expressed by APCs, was rapidly induced by α-GalCer challenge with significantly higher expression in ICG001-treated mice compared to DMSO controls at 1.5 h (Figure S2A in Supplementary Material). There were similar percentages of Ly6C^hi^ and Ly6C^lo^ myeloid cells (CD11b^+^Ly6G^−^) in the liver in DMSO- and ICG001-treated mice, after both control and α-GalCer challenge (Figure S2B in Supplementary Material), suggesting that an increase in local *Il12b* and *Cd40* mRNA expression was likely due to enhanced induction of gene expression in APCs rather than differential numbers of resident (Ly6C^lo^, including Kupffer cells) and recruited inflammatory (Ly6C^hi^) monocyte populations. In contrast to the early effects on IFN-γ responses, liver *Il4* mRNA expression was reduced in ICG001-treated mice compared to DMSO controls at 4.5 h (Figure 4A), accompanied by reduced serum IL-4 at 6 h post α-GalCer challenge, when serum concentrations were elevated above levels in naïve DMSO-treated mice (Figure 4B). Together, these data suggest that β-catenin activity suppresses early α-GalCer-induced IFN-γ responses, while subsequently sustaining the IL-4 response.

### α-GalCer Challenge Suppresses Wnt/β-Catenin Pathway Activity in the Liver

**Figure 5:**
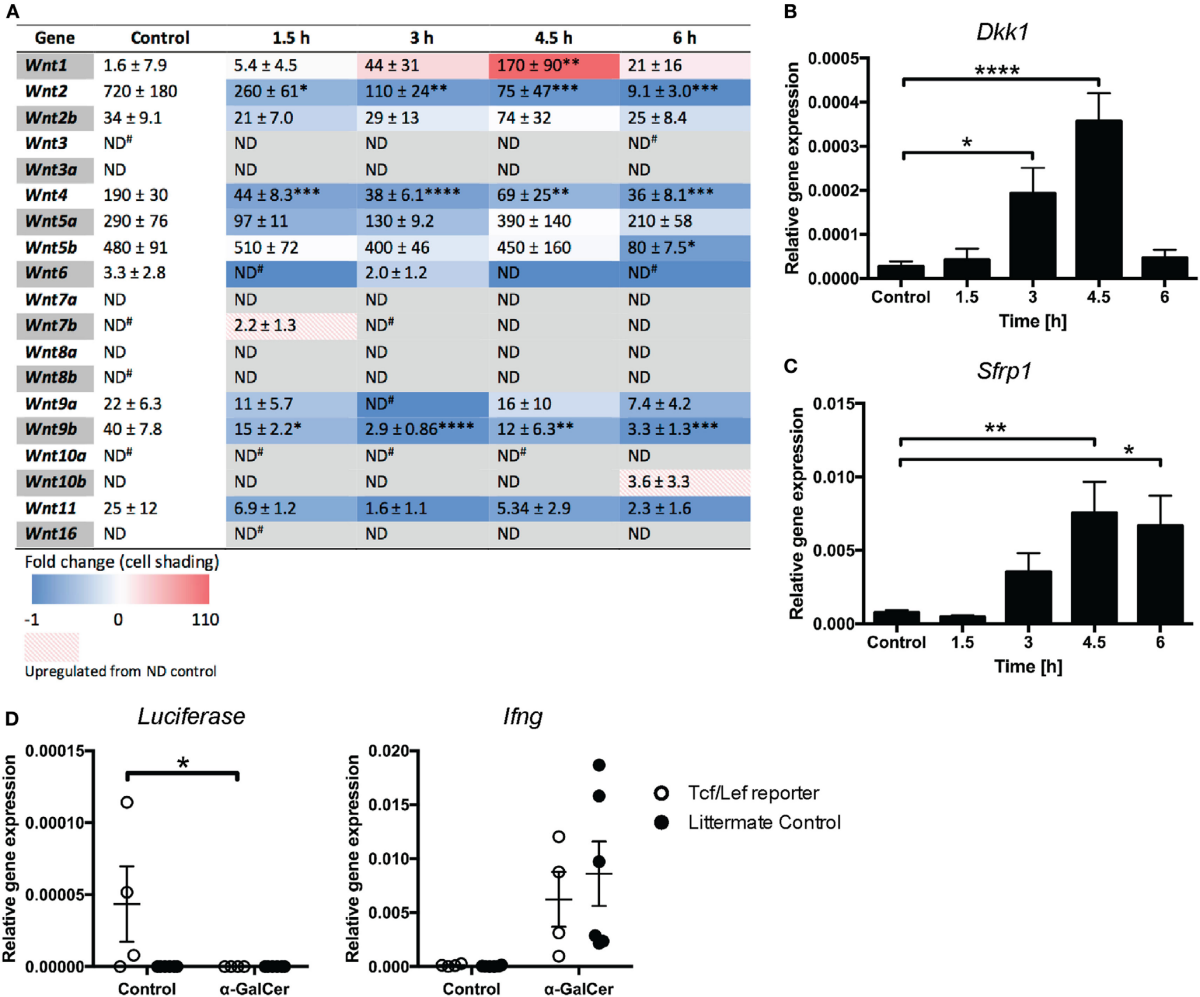
Wnt/β-catenin signaling is suppressed in the liver upon α-galactosylceramide (α-GalCer) challenge. **(A)** Heat map of fold change of Wnt ligand mRNA expression in the liver compared to vehicle control-injected mice determined by quantitative RT-PCR (blue = downregulation, red = upregulation). Numbers within the table represent relative gene expression for individual genes normalized to *Hprt*, expressed as mean (×10^−5^) ± SEM (×10^−5^). The mRNA expression of *Dkk1* **(B)** and *Sfrp1* **(C)** was determined. **(D)** Tcf/Lef TOPFlash mice, expressing luciferase as a reporter of Tcf/Lef transcriptional activity, and transgene-negative littermate controls were challenged with α-GalCer or vehicle control for 6 h. Luciferase expression in liver tissue was determined by quantitative PCR. Expression of *Ifng* was determined as a marker of activation after α-GalCer challenge. Data are means ± SEM of four to nine mice per time point from three **(A–C)** or two **(D)** independent experiments. One-way ANOVA with Dunnett’s **(A–C)** and two-way ANOVA with Bonferroni’s **(D)** correction for multiple comparisons; *p < 0.05, **p < 0.01, ****p < 0.0001.

While β-catenin signaling has been implicated in NKT cell functions (20), a direct role for Wnt ligands has not yet been established. We thus profiled the expression of Wnt ligands in liver tissue of mice challenged with α-GalCer. The rapid induction of cytokine responses (Figure 2) was accompanied by reduced mRNA expression of several Wnt ligands, including *Wnt2, Wnt4*, and *Wnt9b* (Figure 5A), whose expression remained low throughout the 6 h of α-GalCer challenge. *Wnt5b* mRNA expression remained constant initially, but was significantly reduced at 6 h post challenge. By contrast, *Wnt1* was the only ligand whose expression was significantly induced by α-GalCer, peaking at 4.5 h post challenge. In contrast, the expression of *Wnt2b, Wnt5a, Wnt9a*, and *Wnt11* was not significantly altered in liver tissue of α-GalCer challenged mice, and expression of the remaining Wnt ligands was below the detection limit under all conditions (Figure 5A). Remarkably, expression of the endogenous regulators of Wnt signaling, *Dkk1* and *Sfrp1* (Figures 5B,C) was also significantly increased and Tcf/Lef transcriptional activity in liver tissue of β-catenin reporter mice (24) was lost upon α-GalCer challenge (Figure 5D). Comparable α-GalCer-induced *Ifng* expression in the reporter mice and littermate controls demonstrated equal responsiveness to the NKT cell antigen (Figure 5D). Taken together, these data are concordant with active suppression of Wnt/β-catenin signaling in the liver in response to the NKT cell antigen, α-GalCer.

### Temporal Regulation of α-GalCer-Induced IFN-γ Responses by Myeloid Cell-Derived Wnt Ligands

We next sought to establish whether Wnt ligands contribute to the α-GalCer-induced cytokine profiles. APCs are a cellular source of Wnt ligands during homeostasis and inflammation (22, 36). We utilized a mouse model of LysM-Cre-driven *Wls* ablation in myeloid cells, which we have demonstrated to effectively ablate *Wls* expression by F4/80^+^ liver macrophages (7). *Wls* deletion did not affect numbers and location of liver macrophages (7) and CD3^+^NK1.1^+^ NKT cells (Figure S3A in Supplementary Material). While previous studies reported that LEF-1 negatively regulated the transcription of *CD1D* in human cells (18, 19), LysM-Cre-driven *Wls* ablation in mice did not alter expression of *Cd1d* (Figures S3B,C in Supplementary Material).

**Figure 6:**
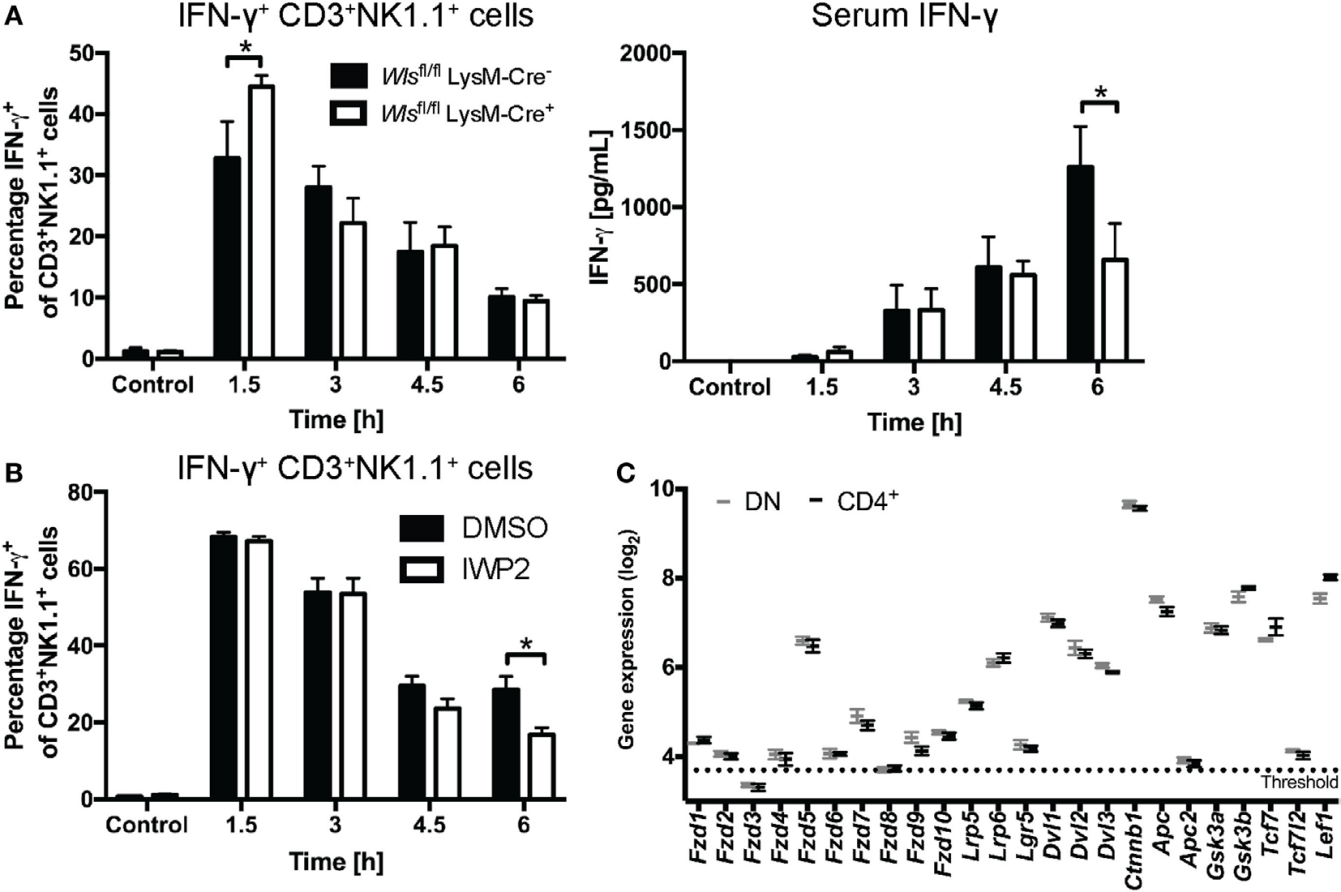
Wnt proteins secreted by myeloid cells upon α-galactosylceramide (α-GalCer) challenge regulate the interferon gamma (IFN-γ) response. **(A)** Mice with a conditional knockout of Wls (*Wls*^fl/fl^ LysM-Cre^+^) and littermate controls (*Wls*^fl/fl^ LysM-Cre^−^) were challenged with α-GalCer. Percentages of CD3^+^NK1.1^+^ natural killer T (NKT) cells expressing IFN-γ in the liver, and the serum levels of IFN-γ were analyzed. Data are means ± SEM of four to six mice per genotype (*n* = 2 for 4.5 h *Wls*^fl/fl^ LysM-Cre^−^ group) per time point cumulatively analyzed in four independent experiments. **(B)** Percentages of CD3^+^NK1.1^+^ NKT cells expressing IFN-γ was determined by flow cytometry in mice treated with DMSO or the Porcupine inhibitor, IWP2. Data are means ± SEM of four to nine mice per treatment for each time point from two to three independent experiments. At each time point, groups in panels **(A,B)** were compared using unpaired two-sided *t*-test; *p < 0.05. **(C)** Mircoarray-based gene expression analyses of sorted CD4^−^ (and CD8-negative, DN) and CD4^+^ liver NKT cells were analyzed for the expression of Wnt signaling components. Data are presented as mean ± SEM. The threshold was set at the expression level of *Il12a*, which is not expressed by NKT cells.

*Wls*^fl/fl^ LysM-Cre^+^ mice exhibited early increased percentages of IFN-γ^+^ CD3^+^NK1.1^+^ cells in the liver compared to littermate controls after α-GalCer challenge (Figure 6A). This phenotype mirrored to some extent the observations made with the β-catenin inhibitor, ICG001 (Figure 3), suggesting that early during the response to α-GalCer, myeloid-derived Wnt proteins drive β-catenin signaling that curbs hepatic IFN-γ responses. By contrast, at 6 h after α-GalCer challenge, IFN-γ concentrations in the serum of *Wls*^fl/fl^ LysM-Cre^+^ mice were significantly lower compared to littermate controls (Figure 6A). As this was not associated with reduced percentages of IFN-γ^+^ CD3^+^NK1.1^+^ (NKT) and CD3^−^NK1.1^+^ (NK) cells in the liver (Figure 6A; Figure S3D in Supplementary Material), regulation of the systemic IFN-γ response at this later time point may occur by Wls-expressing myeloid cells at locations other than the liver or be shaped by cells other than NK and NKT cells. Nevertheless, Wnt ligands from other cellular sources may contribute to NKT cell production of IFN-γ in response to α-GalCer challenge. This view is supported by reduced percentages of IFN-γ^+^ liver CD3^+^NK1.1^+^ cells in mice treated with IWP-2 (37) (Figure 6B), a small molecule inhibitor of the acyltransferase Porcupine, which plays a central role in Wnt secretion (14). In contrast to the impact on IFN-γ, *Wls* deletion in myeloid cells did not affect α-GalCer-induced IL-4 responses in the first 6 h post challenge (data not shown).

While APCs are not only known sources but also targets of Wnt ligands, much less is known about the potential of NKT cells to respond to Wnt signals. We thus assessed whether NKT cells are, in principle, equipped to respond to Wnt ligands. To this end, we performed microarray analyses on α-GalCer-CD1d-tetramer^+^ TCRβ^+^ NK1.1^+^ cells sorted from mouse liver tissue. Indeed, NKT cells express Wnt receptors and co-receptors, β-catenin, components of the β-catenin destruction complex and downstream transcription factors, and there were no apparent differences between the predominant CD4^+^ and CD4^−^ liver NKT cell subsets (Figure 6C; Figure S4 in Supplementary Material). Collectively, these data suggest temporal and differential contributions of Wnt proteins to α-GalCer-induced IFN-γ responses at different stages post challenge, and implicate myeloid cells as a cellular source of Wnt proteins *in vivo* that shapes the functional output of NKT cells.

## Discussion

Studies on contributions of β-catenin activity and Wnt ligands to NKT cell functions *in vivo* have been hampered by the impact of genetic modulation of β-catenin expression on NKT cell development, as well as the limited availability of animal models suitable to assess Wnt functions in the adult organism *in vivo*. Through pharmacologic and conditional genetic targeting of β-catenin activity and key factors that drive Wnt release, collectively the data presented in this study support the conclusions that in response to the NKT cell antigen α-GalCer: (i) Wnt/β-catenin signaling in the liver environment regulates IFN-γ responses; (ii) active suppression of hepatic Wnt/β-catenin signaling shortly after α-GalCer exposure biases cytokine responses toward IFN-γ production; (iii) β-catenin activity independent of APC-derived Wnt ligands contributes to IL-4 expression by NKT cells; (iv) Wnt ligands that act *via* β-catenin-independent signaling contribute to the perpetuation of IFN-γ responses within several hours post challenge; and (v) myeloid-derived Wnt ligands are, in part, responsible for these regulatory effects on NKT cells.

Our findings on the reciprocal contributions of β-catenin to IL-4 and IFN-γ expression by NKT cells are in concordance with a previous report (20) and affirm a role of β-catenin in shaping the functional output from developmentally normal NKT cells. However, in contrast to the assumption that β-catenin-mediated effects on NKT cell functions are the result of Wnt signaling, our data do not support a direct role of Wnt ligands in driving these β-catenin functions in the *in vivo* model of α-GalCer challenge. It is important to note that β-catenin activation is not exclusively driven by Wnt ligands and can, for example, be induced by microbial ligands and hepatic growth factor (38–40). Thus, combined manipulation of both Wnt ligand availability and β-catenin activity offer opportunities for refined insights into the contributions of Wnt/β-catenin signaling in complex *in vivo* settings. Whereas we find it encouraging that ICG001 administration to adult mice showed similar effects on NKT cell IL-4 and IFN-γ expression as genetic ablation (20), obvious limitations of the approach taken here is that ICG001 is likely to impair β-catenin functions in a broad range of cells and only targets β-catenin/CBP interactions. Future studies may focus on cell-specific deletion approaches, preferably inducible in adult tissues, to further underpin the roles of β-catenin in NKT cell functions.

The notion that β-catenin-independent Wnt signaling promotes, whereas Wnt/β-catenin signaling suppresses IFN-γ responses is consistent with the current paradigm of pro- versus anti-inflammatory functions of specific Wnt signaling modalities. For example, in a model of DSS-induced colitis, tamoxifen-induced conditional knockout of Wnt5a resulted in reduced expression of IL-12 and IFN-γ in the colon (41). Neutralizing antibodies against Wnt5a or its receptor Fzd5 impaired antigen-specific IL-12 and IFN-γ responses in PBMC cultures (22). By contrast, Wnt/β-catenin signaling has been attributed immune-modulatory functions *via* the induction of anti-inflammatory, and repression of pro-inflammatory cytokine expression in APCs (36, 40, 42). For example, β-catenin deletion in CD11c^+^ cells impaired pro-inflammatory cytokine responses, including IL-12, in intestinal APCs and enhanced pathology in a colitis model. This was attributed to a shift toward pathogenic Th1 and Th17 cells at the expense of regulatory T cells in the intestine (43). However, it is important to note that β-catenin activity in regulatory T cells impairs their functions and drives colitis and tumor development (44, 45). Moreover, we recently demonstrated that pharmacologic targeting of both β-catenin activity and Wnt production impaired pro-inflammatory cytokine responses in an endotoxemia model in mice (46), indicating pro-inflammatory functions of Wnt/β-catenin signaling in this context. Future studies will focus on identifying individual Wnt/Wnt receptor combinations that shape NKT cell functions and fate, as well as the specific cellular sources of the Wnt ligands that regulate cytokine production by NKT cells. In addition, defining whether Wnt proteins exert their functions through direct signaling in NKT cells or other cells such as APCs, will help delineate the complex temporal contributions of Wnt ligands to NKT cell-mediated immune responses.

Our findings and previous reports emphasize the plethora of Wnt ligands and components of the Wnt signaling machinery that are expressed in liver tissue and are dynamically regulated in response to pathological challenges (7, 35, 47). Repeated exposure to the NKT cell antigen α-GalCer results in enhanced β-catenin signaling in mouse liver tissue, which has been associated with NKT cell anergy in response to chronic activation, and suppresses both IFN-γ and IL-4 production (48). Together with the findings presented here and by others (20), this suggests that transient suppression of Wnt/β-catenin activity after initial challenge, combined with β-catenin-independent Wnt signaling, biases NKT cell functions toward IFN-γ production, while limiting the IL-4 response. Suppression of IFN-γ responses by Wnt/β-catenin signaling would thus be consistent at all stages of NKT cell activation. In this context, it is interesting to note that early during α-GalCer challenge, the majority of the IL-4^+^ CD3^+^ NK1.1^+^ cells also expressed IFN-γ (79.03 ± 1.5% at 1.5 h). Whereas this dual-functionality of IL-4^+^ NKT cells gradually declined (54.3 ± 10.9% at 6 h) neither Porcupine nor β-catenin inhibition affected the percentage of IFN-γ^+^ cells in the IL-4^+^ CD3^+^ NK1.1^+^ population (data not shown). Thus, functionally distinct NKT cell subsets may be selectively responsive to Wnt-mediated regulation of IFN-γ.

The specific molecular mechanisms by which β-catenin may enhance NKT cell-mediated IL-4 responses early during NKT cell activation but limit this response upon chronic exposure remain to be determined. In this context, it is interesting to note that the Wnt ligands whose expression was increased in liver tissue during chronic α-GalCer exposure (*Wnt3a, Wnt5a, Wnt7a, Wnt7b*, and *Wnt10a*) (48), displayed in our study mRNA expression levels below the detection limit (*Wnt3a Wnt7a, Wnt7b, Wnt10a*), or no significant changes in their expression (*Wnt5a*) during the first 6 h following a single α-GalCer injection. This indicates that different subsets of Wnt ligands in the liver microenvironment, in concert with other cytokines and growth factors, may shape NKT cell functions at different stages of activation when compared to anergy. While the specific functional contributions of individual Wnt ligands remain to be established, it is tempting to speculate that this may be reflective of the interaction of NKT cells with different cellular sources of Wnt proteins. There is indeed precedence for differential roles of Wnt proteins in the liver environment depending on the cellular source. Whereas macrophage-derived Wnt proteins promote liver regeneration after partial hepatectomy, Wnt proteins expressed by epithelial cells did not contribute to this process (6).

**Figure 7:**
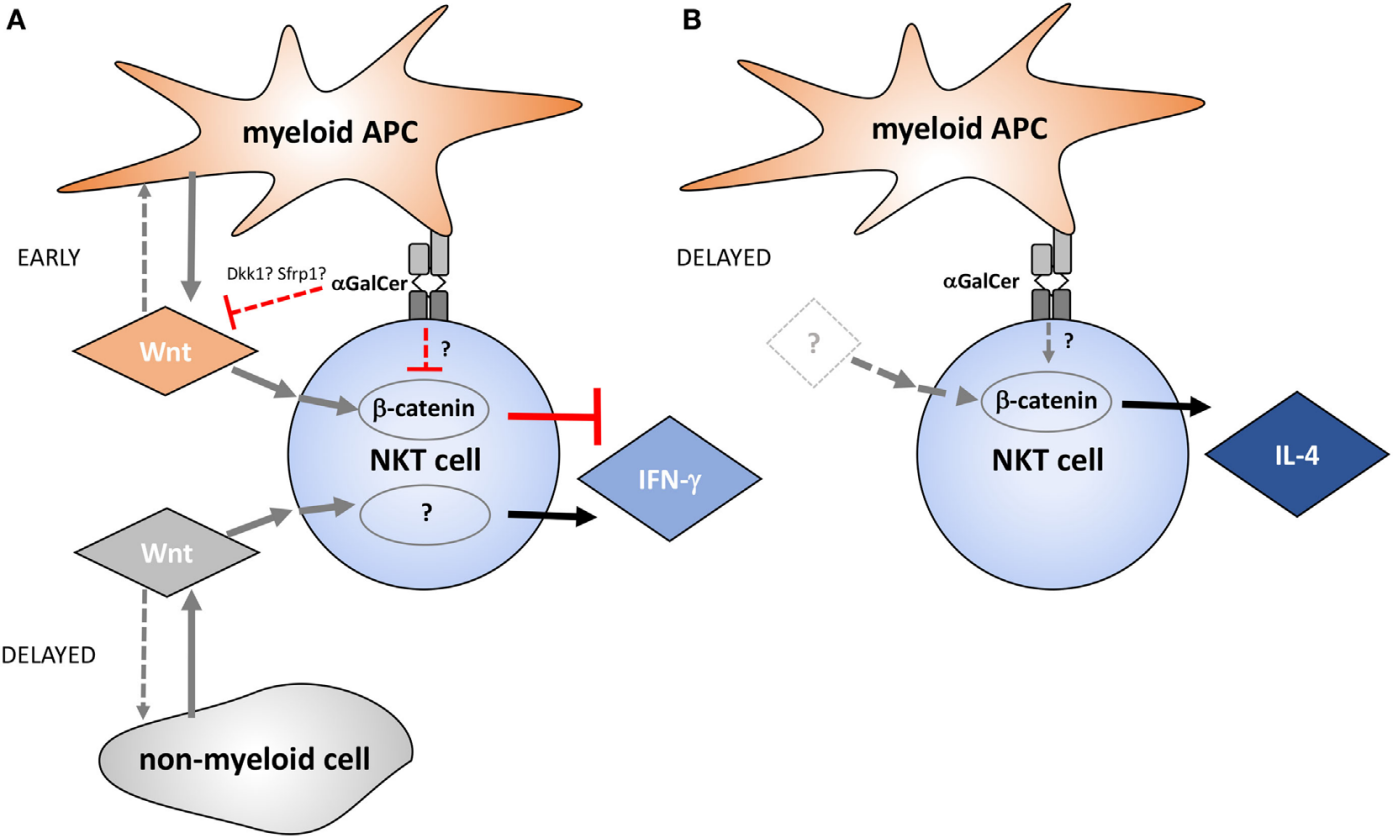
Proposed model of the temporal contributions of Wnt ligands and β-catenin to α-galactosylceramide (α-GalCer)-induced interferon gamma (IFN-γ) and IL-4 responses by liver natural killer T (NKT) cells. **(A)** Wnt production by myeloid cells and Wnt/β-catenin signaling negatively regulate early IFN-γ expression by NKT cells during α-GalCer challenge. Within hours after α-GalCer exposure, Wnt ligand expression in the liver environment is largely suppressed. This is accompanied by increased expression of endogenous Wnt signaling inhibitors such as *Dkk1* and *Sfrp1*, as well as suppression of Tcf/Lef-driven transcriptional activity. Suppression of Wnt/β-catenin signaling favors IFN-γ expression by NKT cells, which is partially supported by β-catenin-independent signaling events driven by Wnt ligands originating largely from non-myeloid cells. **(B)** β-catenin activity contributes to IL-4 expression by liver NKT cells independent of Wnt ligand production, at least during the first 6 h analyzed in the mouse model utilized here. While NKT cells are, in principle, equipped with the signaling machinery required to relay Wnt signaling *via* β-catenin-dependent and -independent pathways as depicted in this model, direct experimental evidence for contributions of these molecular networks in NKT cells is pending. Moreover, it is important to emphasize that the effects of Wnt pathway perturbation on NKT cell cytokine responses may also result from indirect effects of Wnt ligands on other cells, such as antigen-presenting cells (APCs), NK cells, and hepatocytes.

Taken together, the data presented in this study implicate Wnt proteins as regulators of NKT cell functionality in the liver environment (Figure 7). Several small molecule inhibitors that target Wnt production and β-catenin activity are currently in clinical trials (49). NKT cells not only have pathogenic roles, such as in non-alcoholic fatty liver disease and toxin-induced hepatitis (50), but also exert protective functions, for example, due to IFN-γ-mediated repression of hepatitis B virus replication in hepatocytes (51). Thus, detailed understanding of the roles of Wnt proteins and β-catenin in defining NKT cell functions in health and disease may not only be exploited therapeutically by targeted skewing of NKT cell functions but may also reveal potential for adverse outcomes of pharmacologic Wnt pathway perturbation.

## Ethics Statement

All procedures involving animals adhered to the guidelines of the National Health and Medical Research Council Australian Code for the Care and Use of Animals for Scientific Purposes, and were approved by the Animal Ethics Committee of The University of Queensland (UQDI/571/12; UQDI/554/15) and the University of Melbourne Animal Ethics Committee (06089).

## Author Contributions

JK, MJ, LP, JM, TT-T, LT, TN, and DM performed experiments. JK, MJ, LP, DM, SB, AGB, DG, and AB designed experiments. JK and AB analyzed the data. RV, RS, and KK provided reagents and protocols. JK and AB wrote the manuscript, and all authors provided editorial input.

## Conflict of Interest Statement

The authors declare that the research was conducted in the absence of any commercial or financial relationships that could be construed as a potential conflict of interest.

## Supplementary Material

Figure S1 NK cell- and T cell-derived interferon gamma (IFN-γ). The percentage of CD3^+^NK1.1^−^ T cells **(A)** and CD3-NK1.1^+^ NK cells **(B)** in the liver expressing IFN-γ in response to α-galactosylceramide (α-GalCer) challenge was determined by flow cytometry. Expression of IL-4 by CD3^+^NK1.1^−^ T cells **(C)** and CD3^−^NK1.1^+^ NK cells **(D)** in the liver was determined. Data are means ± SEM of four to seven mice per time point analyzed cumulatively in two independent experiments. Groups were compared by one-way ANOVA with Dunnett’s correction for multiple comparisons; **p* < 0.05, ***p* < 0.01, ****p* < 0.001, *****p* < 0.0001.

Figure S2 Characterization of myeloid cell populations in the liver. Mice were treated with DMSO or ICG001 prior to challenge with α-galactosylceramide (α-GalCer) for 1.5 h. **(A)** The percentage of Ly6C^hi^ and Ly6C^lo^ monocytes (CD11b^+^Ly6G^−^) in the liver was determined. **(B)** The liver mRNA expression of *Cd40*, a costimulatory molecule expressed on antigen-presenting cells was determined by quantitative PCR (***p* < 0.01). Data are means ± SEM of four to nine mice per treatment for each time point analyzed in two to three independent experiments. Groups were compared by **(A)** two-way ANOVA with Bonferroni’s correction for multiple comparisons; **(B)** unpaired two-sided *t* test; ***p* < 0.01.

Figure S3 Characterization of conditional Wls knockout mice. **(A)** The percentages of CD3^+^NK1.1^+^ natural killer T cells in the liver were determined by flow cytometry. **(B)** The mRNA expression of *Cd1d* was determined in liver tissue after α-galactosylceramide challenge in mice with a conditional knockout of Wls (*Wls*^fl/fl^ LysM-Cre^+^) and their littermate controls (*Wls*^fl/fl^ LysM-Cre^−^). **(C)** Bone marrow-derived macrophages were cultured for 6 days in the presence of L cell-conditioned medium. mRNA expression of *Cd1d* was determined by qPCR. Data are means ± SEM of cultures from 7 Cre^−^ and 10 Cre^+^ individual mice. **(D)** The percentages of CD3^−^NK1.1^+^ NK cells in the liver expressing interferon gamma (IFN-γ) were determined by flow cytometry. Data in panels **(A,B,D)** are from *n* = 4–6 mice per genotype for each time point (*n* = 2 for 4.5 h *Wlsfl/fl* LysM^−^Cre^−^ group) from four independent experiments. Groups were compared by **(A,C)** unpaired two-tailed *t* test; **(B,D)** two-way ANOVA with Bonferroni’s correction for multiple comparisons.

Figure S4 Surface expression of Fzd1 and Fzd7 on CD3^+^NK1.1^+^ natural killer T cells. Flow cytometry was performed to confirm surface expression of Fzd1 and Fzd7 on CD3^+^NK1.1^+^ cells. Data representative of eight mice.

## Abbreviations

α-GalCer, α-galactosylceramide; APC, antigen-presenting cell; Dkk, Dickkopf; Fzd, Frizzled; IFN-γ, interferon gamma; IL, interleukin; Lef, lymphoid enhancing factor; LysM, lysozyme M; NKT cell, natural killer T cell; Sfrp, soluble Frizzled-related protein; Tcf, T cell factor; Wls, Wntless.
