# Supplementary figures and images for: Temporal Regulation of Natural Killer T Cell Interferon Gamma Responses by β-Catenin-Dependent and -Independent Wnt Signaling
Source: Front Immunol. 2018 Mar 16;9:483. doi: 10.3389/fimmu.2018.00483 (PMC5864864; doi:10.3389/fimmu.2018.00483)

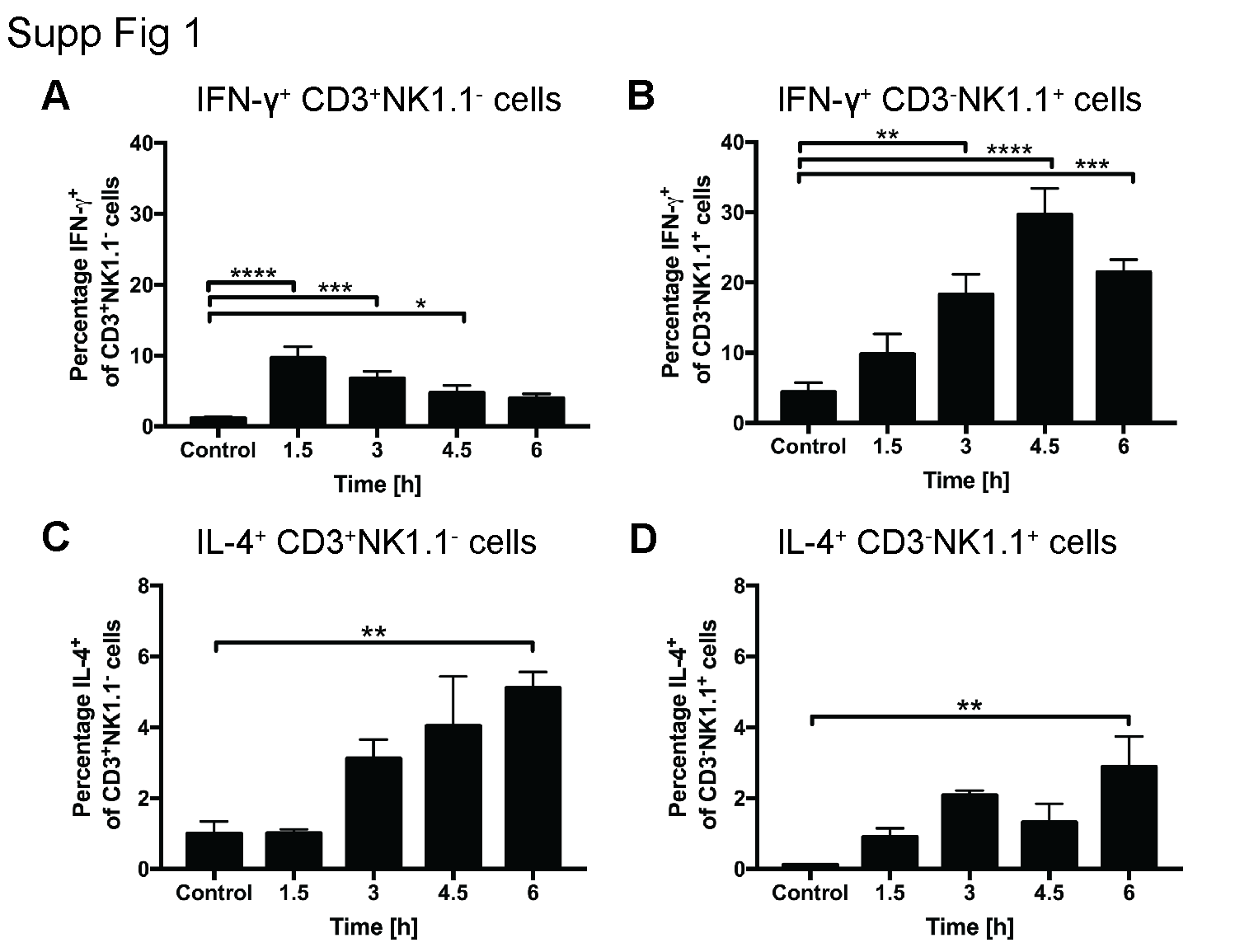

Supplement: Figure S1 — NK cell- and T cell-derived interferon gamma (IFN-γ). The percentage of CD3+NK1.1− T cells (A) and CD3-NK1.1+ NK cells (B) in the liver expressing IFN-γ in response to α-galactosylceramide (α-GalCer) challenge was determined by flow cytometry. Expression of IL-4 by CD3+NK1.1− T cells (C) and CD3−NK1.1+ NK cells (D) in the liver was determined. Data are means ± SEM of four to seven mice per time point analyzed cumulatively in two independent experiments. Groups were compared by one-way ANOVA with Dunnett’s correction for multiple comparisons; *p < 0.05, **p < 0.01, ***p < 0.001, ****p < 0.0001. [file Image_1.tif]

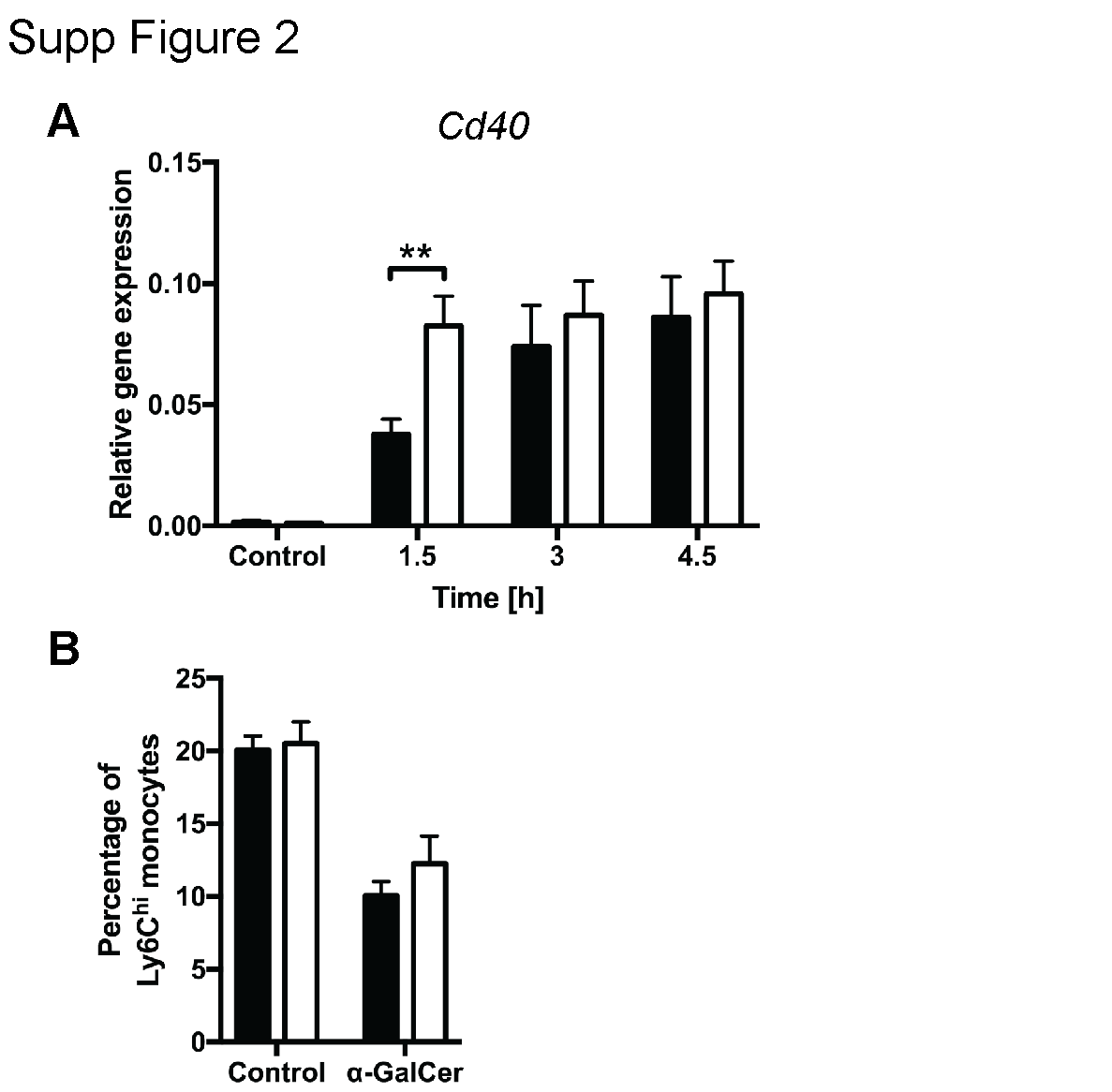

Supplement: Figure S2 — Characterization of myeloid cell populations in the liver. Mice were treated with DMSO or ICG001 prior to challenge with α-galactosylceramide (α-GalCer) for 1.5 h. (A) The percentage of Ly6Chi and Ly6Clo monocytes (CD11b+Ly6G−) in the liver was determined. (B) The liver mRNA expression of Cd40, a costimulatory molecule expressed on antigen-presenting cells was determined by quantitative PCR (**p < 0.01). Data are means ± SEM of four to nine mice per treatment for each time point analyzed in two to three independent experiments. Groups were compared by (A) two-way ANOVA with Bonferroni’s correction for multiple comparisons; (B) unpaired two-sided t test; **p < 0.01. [file Image_2.tif]

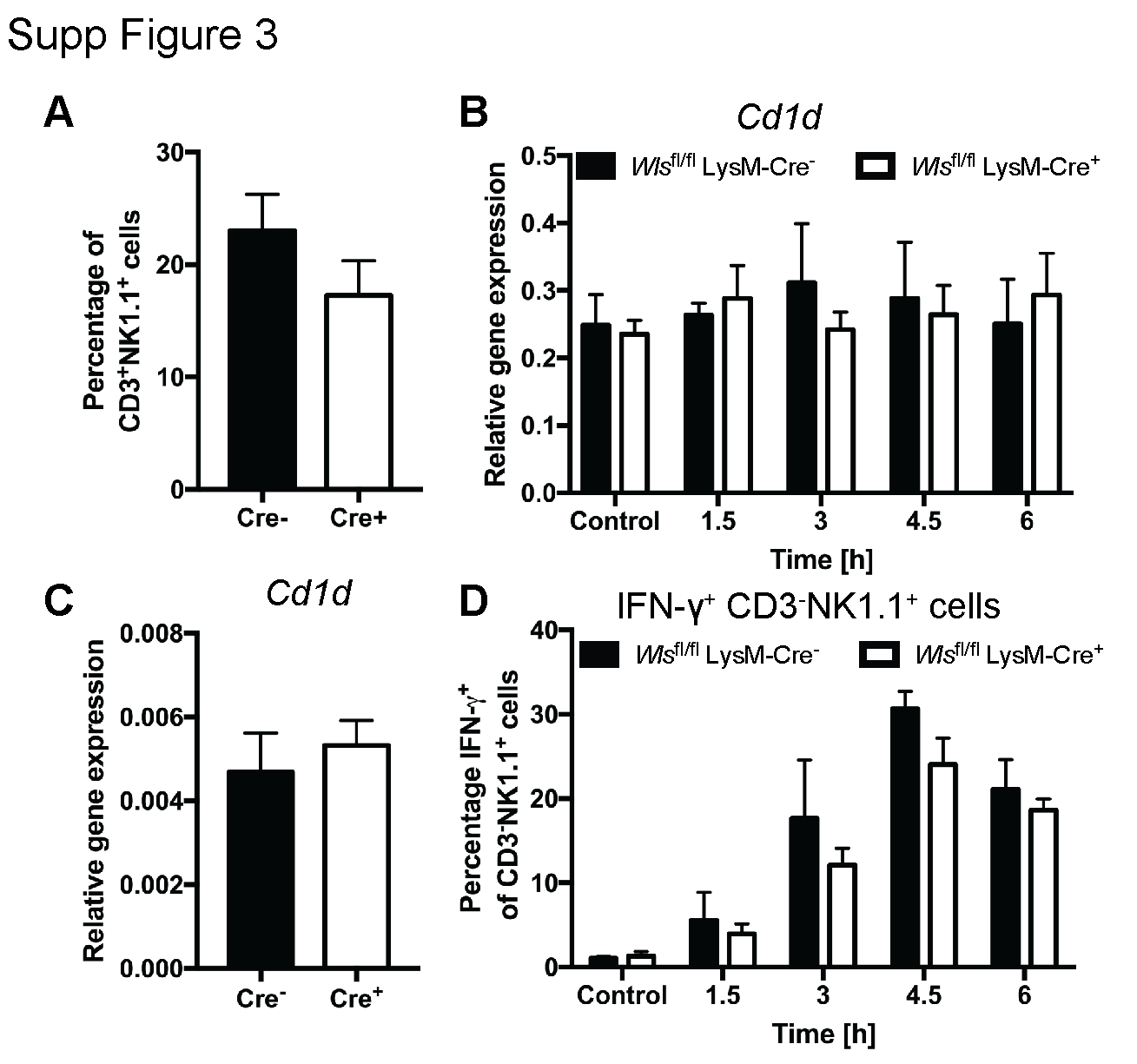

Supplement: Figure S3 — Characterization of conditional Wls knockout mice. (A) The percentages of CD3+NK1.1+ natural killer T cells in the liver were determined by flow cytometry. (B) The mRNA expression of Cd1d was determined in liver tissue after α-galactosylceramide challenge in mice with a conditional knockout of Wls (Wlsfl/fl LysM-Cre+) and their littermate controls (Wlsfl/fl LysM-Cre−). (C) Bone marrow-derived macrophages were cultured for 6 days in the presence of L cell-conditioned medium. mRNA expression of Cd1d was determined by qPCR. Data are means ± SEM of cultures from 7 Cre− and 10 Cre+ individual mice. (D) The percentages of CD3−NK1.1+ NK cells in the liver expressing interferon gamma (IFN-γ) were determined by flow cytometry. Data in panels (A,B,D) are from n = 4–6 mice per genotype for each time point (n = 2 for 4.5 h Wlsfl/fl LysM−Cre− group) from four independent experiments. Groups were compared by (A,C) unpaired two-tailed t test; (B,D) two-way ANOVA with Bonferroni’s correction for multiple comparisons. [file Image_3.tif]

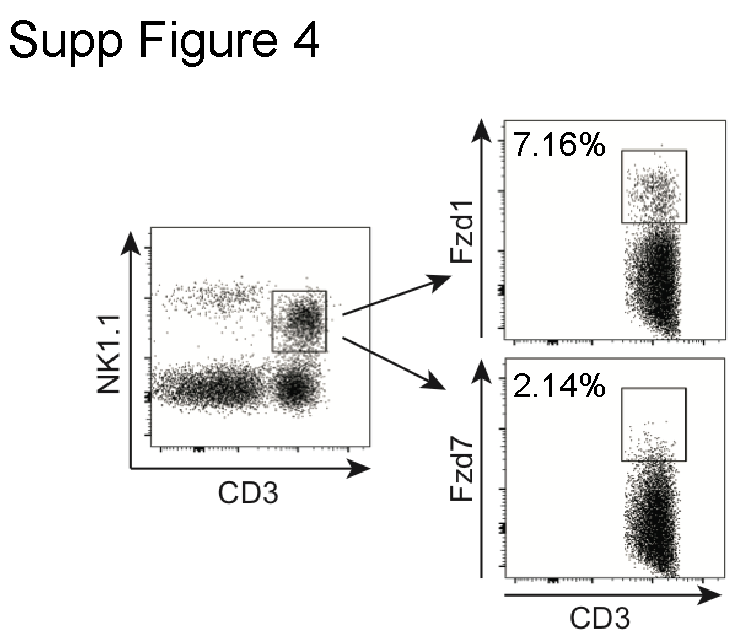

Supplement: Figure S4 — Surface expression of Fzd1 and Fzd7 on CD3+NK1.1+ natural killer T cells. Flow cytometry was performed to confirm surface expression of Fzd1 and Fzd7 on CD3+NK1.1+ cells. Data representative of eight mice. [file Image_4.tif]
